# Federated Learning with Privacy Preserving for Multi- Institutional Three-Dimensional Brain Tumor Segmentation

**DOI:** 10.3390/diagnostics14242891

**Published:** 2024-12-23

**Authors:** Mohammed Elbachir Yahiaoui, Makhlouf Derdour, Rawad Abdulghafor, Sherzod Turaev, Mohamed Gasmi, Akram Bennour, Abdulaziz Aborujilah, Mohamed Al Sarem

**Affiliations:** 1 Mathematics, Informatics and Systems LAboratory—LAMIS Laboratory, University of Echahid Cheikh Larbi Tebessi, Tebessa 12000, Algeria; yahiaoui.mohammedelbachir@univ-tebessa.dz (M.E.Y.); mohamed.gasmi@univ-tebessa.dz (M.G.); 2 Artificial Intelligence and Autonomous Things Laboratory—LIAOA, University of Oum el Bouaghi, Oum El Bouaghi 04000, Algeria; 3 Faculty of Computer Studies (FCS), Arab Open Universit—Oman, Muscat 130, Oman; rawad.a@aou.edu.om; 4 Department of Computer Science and Software Engineering, College of Information Technology, United Arab Emirates University, Al Ain 15551, United Arab Emirates; 5 Department of Management Information System, College of Commerce & Business Administration, Dhofar University, Salalah 211, Oman; aaborujilah@du.edu.om; 6 Department of Information Technology, Aylol University College, Yarim 547, Yemen; mohsarem@gmail.com

**Keywords:** brain tumor segmentation, 3D U-Net, federated learning, privacy-preserving

## Abstract

Background and Objectives: Brain tumors are complex diseases that require careful diagnosis and treatment. A minor error in the diagnosis may easily lead to significant consequences. Thus, one must place a premium on accurately identifying brain tumors. However, deep learning (DL) models often face challenges in obtaining sufficient medical imaging data due to legal, privacy, and technical barriers hindering data sharing between institutions. This study aims to implement a federated learning (FL) approach with privacy-preserving techniques (PPTs) directed toward segmenting brain tumor lesions in a distributed and privacy-aware manner.Methods: The suggested approach employs a model of 3D U-Net, which is trained using federated learning on the BraTS 2020 dataset. PPTs, such as differential privacy, are included to ensure data confidentiality while managing privacy and heterogeneity challenges with minimal communication overhead. The efficiency of the model is measured in terms of Dice similarity coefficients (DSCs) and 95% Hausdorff distances (HD95) concerning the target areas concerned by tumors, which include the whole tumor (WT), tumor core (TC), and enhancing tumor core (ET). Results: In the validation phase, the partial federated model achieved DSCs of 86.1%, 83.3%, and 79.8%, corresponding to 95% values of 25.3 mm, 8.61 mm, and 9.16 mm for WT, TC, and ET, respectively. On the final test set, the model demonstrated improved performance, achieving DSCs of 89.85%, 87.55%, and 86.6%, with HD95 values of 22.95 mm, 8.68 mm, and 8.32 mm for WT, TC, and ET, respectively, which indicates the effectiveness of the segmentation approach, and its privacy preservation.Conclusion: This study presents a highly competitive, collaborative federated learning model with PPTs that can successfully segment brain tumor lesions without compromising patient data confidentiality. Future work will improve model generalizability and extend the framework to other medical imaging tasks.

## 1. Introduction

Brain tumors are abnormal cellular growths in the brain, classified as benign or malignant, resulting from genetic factors, environmental influences, and radiation exposure [1]. Their complexity lies in the diversity of tumor types and their response to treatment, requiring accurate diagnosis for effective management, which may involve surgery, radiotherapy, chemotherapy, or targeted therapies. Imaging techniques such as magnetic resonance imaging (MRI) and computed tomography (CT) are essential for visualizing tumor features, with MRI offering superior soft-tissue contrast. However, manual diagnosis poses problems due to subjective interpretation and the variability between radiologists, complicating the delineation of tumor boundaries [2]. These challenges highlight the need for innovative solutions such as artificial intelligence (AI), which automates the analysis of imaging data. AI algorithms, particularly DL models, improve diagnostic accuracy by rapidly processing large datasets and identifying patterns that often elude human observers. In addition, DL can standardize diagnoses across facilities, reducing interobserver variability and improving overall diagnostic consistency.

According to these studies [3,4,5], the effectiveness of these models is contingent upon the availability of extensive and diverse medical imaging data for robust training. However, acquiring such data, particularly in medical imaging, presents a considerable challenge. The intricacies of labeling medical image data, as highlighted by [6,7,8,9], require specialized knowledge, and the scarcity of labeled datasets constitutes a major bottleneck for the development and optimization of DL models.

To address these challenges, collaborative initiatives across institutions appear promising. By pooling resources and expertise, such collaborations can potentially overcome data limitations and enhance the deep models’ results across diverse medical imaging datasets. However, the conventional centralization of medical data in a single location faces many obstacles, including legal and privacy concerns, technical complexities, and data ownership issues. These challenges are even greater in the context of international collaborations, where navigating diverse regulatory frameworks and ensuring data security are crucial.

Recognizing that FL alone may be insufficient, especially due to the risk of reconstruction of training examples by model inversion attacks, we focus on PPTs in this setting. Using a deep 3D U-Net model and the BraTS 2020 dataset, our study aims to demonstrate the potential of FL combined with PPTs to facilitate secure, collaborative, and efficient medical image analysis.

In light of the pressing need for accurate brain tumor segmentation, particularly given the complexities of diagnosing and treating such conditions, our research introduces an FL approach that addresses the critical challenges of data privacy and sharing across multiple institutions. This study makes several key contributions: first, it pioneers the application of FL in the medical domain specifically for brain tumor segmentation; second, it implements advanced PPTs, including differential privacy, to ensure the robust protection of individual patient data; third, it explores secure model aggregation methods in a heterogeneous and distributed environment; fourth, it simulates real-world healthcare scenarios to validate the feasibility and effectiveness of the proposed approach; and, finally, it integrates comprehensive privacy measures to uphold ethical standards and ensure compliance. Collectively, these contributions not only enhance the accuracy of brain tumor segmentation but also establish a framework for future research in secure and collaborative medical image analysis. The main goal of this work was to explore and implement FL techniques for brain tumor segmentation while ensuring robust privacy protection for sensitive medical data. By leveraging a deep 3D U-Net model on the BraTS 2020 dataset, we aimed to demonstrate the feasibility and effectiveness of collaborative model training across multiple institutions without the need for centralized data sharing. This approach not only addresses the challenges due to data scarcity and privacy concerns in medical imaging but also seeks to enhance diagnostic accuracy and improve patient outcomes through secure and efficient analysis of brain tumor images.

The remainder of this article is organized as follows: In Section 2, we first review the relevant literature. Section 3 introduces some background knowledge. Section 4 delves into the details of our methodology. Section 5 presents the obtained results, and we discuss the implications and significance of our findings. In Section 6, we conclude this paper.

## 2. Related Work

Many studies have predominantly adopted centralized approaches for machine learning (ML) tasks [10,11,12,13,14,15,16,17,18,19,20,21]. A paper [19] explores advanced hybrid techniques for the early detection of brain tumors based on ML and DL models. It presents four systems: the first combines ANN and FFNN using LBP, co-occurrence matrix (GLCM), and transform (DWT); the second uses pretrained GoogLeNet and ResNet-50 models for feature extraction and classification; the third merges (CNN) with (SVM) to improve classification; and the fourth combines GoogLeNet, ResNet-50, and handcrafted features. In that study, the FFNN model achieved an accuracy of 99.9%. A study [20] used a dataset of 3060 MRI images divided into four classes: three malignant types and one normal class. The proposed hybrid system combined AlexNet + SVM and achieved excellent performance with an accuracy of 95.10%, a sensitivity of 95.25% and a specificity of 98.50%. A paper [21] presents a study on the diagnosis of intracranial hemorrhage using advanced imaging techniques, focusing on CT images. Three methods are presented. The first uses pretrained CNNs such as GoogLeNet, ResNet-50 and AlexNet, achieving accuracies of 94%, 91.7%, and 91.5%, respectively. The second method integrates CNNs with SVM, achieving accuracies of 97.4%, 97.2% and 95.7%. The third method uses an ANN that combines the features extracted from CNNs with the features from the co-occurrence matrix (GLCM and LBP), achieving the highest accuracy of 99.3%. This conventional paradigm involves aggregating and processing data in a centralized manner, often posing challenges related to data privacy, security, and scalability. Recently, FL has demonstrated itself to be a paradigm with the potential to transform the field of ML, offering a decentralized approach that is particularly well suited to the distributed nature of modern computing. In FL, the training of models is conducted locally on distributed devices, such as mobile phones or edge devices. Only aggregated model updates are shared with a central server. This enables collaborative model training while mitigating the privacy concerns associated with centralized data storage. Several studies have explored the application of FL in the healthcare domain, particularly in the context of medical image segmentation and tumor analysis. For instance, Sheller et al. [22] demonstrated the feasibility of using FL to identify cancerous disease cells in the brain across different medical institutions leaving no doubt about the usefulness of FL, which improves the performance of the intelligent model without compromising and sharing the data of the patients involved. Similarly, Qiu et al. [23] used a federated semisupervised learning (FSSL) model for medical image segmentation, which incorporates a federated pseudo-labeling strategy to address the annotation deficiency for unlabeled clients. In [24], the authors present a new approach to brain tumor segmentation using FL, which enables collaborative model learning without the need to share sensitive patient data. The study used a 3D U-Net architecture on the BraTS 2020 dataset, achieving high segmentation performance with a tumor-wide DSC of 0.896 and an HD95 of 23.611 mm, comparable to those of centralized models. In the multiorgan segmentation context, Xie et al. [25] proposed an FL approach that leverages inconsistent labels across institutions to improve model robustness and generalization (Fed-MENU). This method utilizes a multiencoding U-Net to extract organ-specific features from partially labeled data and was evaluated with six public abdominal CT datasets. Additionally, Liu and co-authors [26] introduced episodic learning in continuous frequency space (ELCFS) to address the problem of federated domain generalization (FedDG) in medical image segmentation. Their approach involves transferring distribution information across clients in a simple privacy-protecting manner and implementing a boundary-oriented episodic learning paradigm to enhance model generalization. FL has successfully ensured data privacy and collaborative model training in fields beyond medical imaging. Examples include its application in intrusion detection systems (IDSs) [27,28], the development of smart cities [29], and electrical driving systems [30]. However, despite these successes, the adoption of FL with advanced PPTs remains limited. Presently, PPTs in FL have garnered significant attention due to the increasing focus on safeguarding sensitive information during collaborative model training. Several works [31,32,33,34,35] have explored diverse privacy-preserving (PP) mechanisms, aiming to strike a balance between model accuracy and individual privacy. For instance, Chen et al. [31] proposed an efficient PP and traceable FL framework with minimal overhead. The approach incorporates hierarchical aggregation, random seed-based noise addition, and encryption for secure global parameter transmission through subaggregators. Authors [32] proposed PrivateKT for PP knowledge transfer in FL. The method demonstrates comparable performance to centralized learning while providing strict privacy guarantees through differential privacy constraints. Shao et al. [33] developed a selective knowledge-sharing mechanism for federated distillation, which has the potential to enable a PP, communication-efficient, and heterogeneity-adaptive federated training framework. Several works rely on blockchain-based FL [36,37,38]. In particular, in [36], the authors propose a 5G framework integrating blockchain for enhanced security and privacy. The system incorporates a DRL optimization strategy, local noise addition for privacy, and a DPoS protocol for efficient transaction validation and edge server computation. Similarly, a study [37] proposed an Industrial Internet of Things BCFL model with adaptive differential privacy, using the Laplace mechanism to introduce noise during model updates. The model adapts the cropping threshold dynamically, minimizing noise’s impact on accuracy, and incorporates validation and consensus mechanisms to detect and prevent malicious attacks, ensuring fairness through a reputation-based node assessment system. In one recent example [38], the authors explored privacy solutions in the context of blockchain-enabled federated learning (BCFL), summarizing background information, evaluating integration architectures, addressing privacy concerns, and highlighting applications and challenges in this emerging field. In the medicine and healthcare domain, Li et al. Ref. [34] explored FL for brain tumor segmentation while preserving patient data privacy. They implemented and evaluated practical systems on the BraTS 2018 dataset, investigating the use of differential privacy techniques. The study compared federated averaging algorithms, addressing momentum-based optimization and imbalanced training nodes. Additionally, the authors empirically studied the sparse vector technique to provide a strong differential privacy guarantee, highlighting the trade- off between model performance and privacy protection. Authors [35] introduced PriMIA (privacy-preserving medical image analysis), a framework for secure medical image analysis. PriMIA employs FL and encrypted diagnosis to protect patient privacy while ensuring accurate classification. The study evaluated the framework’s security against model inversion attacks. In the literature [39], a comprehensive overview of current and future approaches to PP artificial intelligence, particularly in medical imaging, is provided. The authors addressed the challenges, potential vulnerabilities compromising privacy, and future developments in the field. Moreover, a paper [40] proposes a mobile crowdsensing system that leverages unmanned aerial vehicles (UAVs), incorporating local differential privacy and a reinforcement-learning-based incentive system. In [41,42], homomorphic encryption was employed in FL to reinforce the security of parameters shared with the external network system, ensuring continued privacy of patient data and facilitating the accurate aggregation of models. From our literature review, we identified several important limitations in previous studies. Many studies have primarily used centralized approaches for ML tasks, raising significant concerns related to data confidentiality, security, and scalability. Challenges in acquiring sufficient medical imaging data, due to legal, technical, and privacy-related obstacles, were also common, particularly in multi-institutional collaborations. While FL has emerged as a promising solution, its application to medical imaging, especially for brain tumor segmentation, remains limited. Furthermore, existing research often overlooked advanced PPTs, which are crucial for protecting sensitive patient data during collaborative learning. Our study addresses these gaps by introducing a specifically designed FL framework for brain tumor segmentation using a deep 3D U-Net model on the BraTS 2020 dataset. We implemented privacy techniques, including differential privacy, to ensure robust protection of individual patient data throughout the collaborative learning process. In addition, we explored secure model aggregation methods in a heterogeneous and distributed environment, enhancing privacy while improving model performance. In summary, our work not only fills existing gaps in the literature by combining FL with advanced privacy protection measures but also makes a significant contribution to the field of secure and efficient medical image analysis.

## 3. Background

### 3.1. Neural Network Architecture

**Figure 1:**
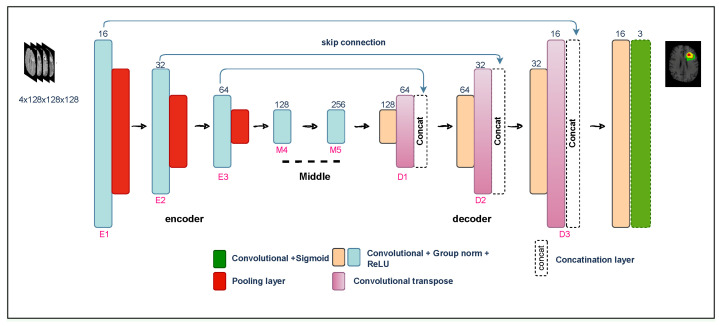
Overview of proposed neural network architecture.

In addressing the task of segmenting distinct Glioma subregions within 3D medical images, our proposed neural network architecture draws inspiration from the U-Net framework. Comprising a dual-path structure, the model conducts its analysis with a 3D input tensor of shape (4, 128, 128, 128), initiating a comprehensive exploration. The contracting path employs convolutional blocks, integrating group normalization and ReLU activations for enhanced nonlinearity, followed by pooling operations to capture contextual information, where each convolutional block applies two 3D convolutions with a kernel size of 3 × 3 × 3. Progressing through additional convolutional blocks, the contracting path gradually augments filters to extract high-level features and downsample the input volume. Transitioning to the intermediate stage, the model introduces further convolutional blocks (M4 and M5). It utilizes transposed convolutions (Conv3DTranspose) for upsampling and introduces innovative concatenation blocks in the expanding path for seamless communication between paths. Refinement in the expanding path involves convolutional blocks to restore spatial resolution, culminating in the final segmentation map through a 1 × 1 × 1 convolutional layer with a sigmoid activation function. Figure 1 provides a visual representation of this tailored architecture.

### 3.2. Dataset

**Figure 2:**
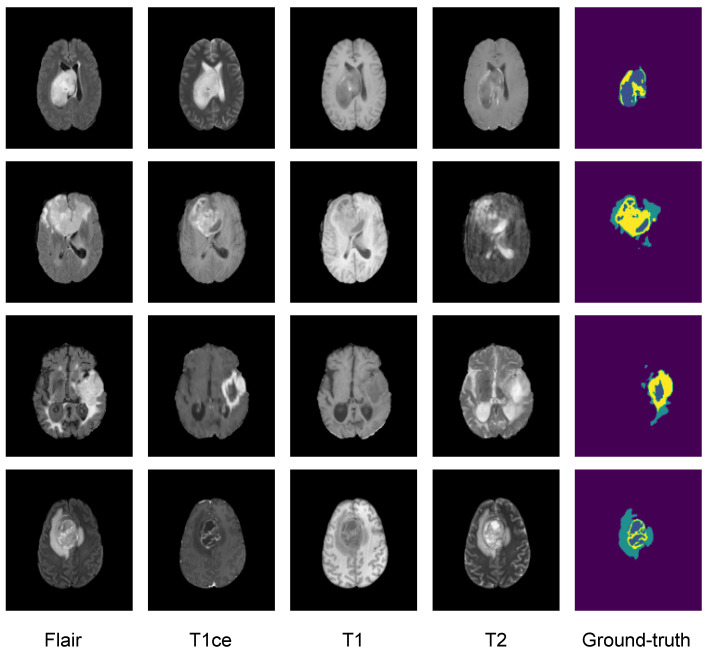
Sample glioma from the BraTS 2020 training dataset. Yellow: enhancing tumor (ET), blue: nonenhancing tumor/necrotic tumor (NET/NCR), green: peritumoral edema (ED).

In this study, we evaluated the performance of the proposed intelligent model on the BraTS2020 dataset [42,43,44,45], which is well known in the scientific community and freely available to the public. It contains four distinct MRI brain scans from glioma patients: the native (T1), post contrast T1-weighted (T1Gd), T2-weighted (T2), and T2 fluid attenuated inversion recovery (T2-FLAIR) volumes, each paired with a ground truth mask, all from multiple institutional sources. The masks highlight three key labels: peritumoral edema (ED-label 2), the necrotic and nonenhancing tumor core (NCR/NET label 1), and ET (label 4). The dataset is challenging due to the variability in imaging protocols, scanner types, and patient populations across institutions. We trained and tested our model on the BraTS2020 training dataset, which was divided into subsets, with 267 cases for training, 66 for validation, and 36 for testing. Figure 2 illustrates the format of a multimodality brain MRI scan together with an example of the corresponding expert annotation

### 3.3. Image Preprocessing

To construct a coherent input for the neural network, a multicontrast MRI stack was created by combining four distinct MRI sequences. In the initial stages of preprocessing, a meticulous approach was taken to normalize the intensities of each MRI sequence independently within a specified range. This involved clipping intensities to the 1st and 99th percentiles of the nonzero voxels distribution of the volume, followed by min–max normalization. Subsequently, the images underwent a series of spatial adjustments. Initially, the images were cropped to a variable size, ensuring the smallest bounding box encapsulating the entire brain. As a further refinement, random re-ropping was applied to achieve a consistent patch size of 128 × 128 × 128.

### 3.4. Data Augmentation Techniques

To enhance the generalization capabilities of both the centralized model and the FL model, we employed a variety of data augmentation techniques. Notably, for the FL model, each of the four clients had distinct random data augmentation applied to their local models, ensuring diversity in the training process. These augmentations were executed for each epoch and each observation (patient data) with respective probabilities of application inspired by [43]. The data augmentation techniques included

- Input Channel Rescaling: A factor within the range of 0.9 to 1.1 was multiplied to each voxel, with a probability of 80%.
- Input Channel Intensity Shift: A constant within the range of −0.1 to 0.1 was added to each voxel, with a probability of 10%.
- Additive Gaussian Noise: A random noise was generated using a centered normal distribution with a standard deviation of 0.1 and added to the input data.
- Input Channel Dropping: With a 16% chance, one of the input channels had all its voxel values randomly set to zero.
- Random Flip Along Each Spatial Axis: The data were subjected to a random horizontal flip, a random vertical flip, and potentially a random flip along the depth, with a probability of 80%.

### 3.5. Loss Function

In our research, a customized loss function was designed to train the neural network effectively, based on the Dice loss [44], a widely acknowledged metric renowned for its efficacy in medical image segmentation tasks. This loss was computed on a per-batch and per-channel basis, maintaining simplicity without introducing specific weightings to optimize the neural network weights. it is given by

(1) $$DSC=1-\frac{1}{N}\sum_{n}\frac{S_{n}\times R_{n}+\varepsilon }{{S_{n}}^{2}+{R_{n}}^{2}+\varepsilon }$$

where *N* is the total number of classes; $S_{n}$ and $R_{n}$ are the prediction and ground truth for each channel. A smoothing factor (ε) equal to 1 was incorporated to improve stability. Unlike traditional approaches, our optimization strategy targeted the final tumor regions of interest (ET, TC, and WT), bypassing the individual components (such as NET-NCR and ED). The neural network’s output, structured as a 3-channel volume, provided distinct probability maps for each tumor region.

### 3.6. Evaluation Metrics

The evaluation metrics used in this study encompassed a range of important measures, including the DSC, sensitivity, specificity, and HD95. This allowed for an overall assessment of the performance of the models in medical segmentation tasks, capturing aspects such as overlap, true positive rate, true negative rate, and boundary dissimilarity. Each metric offers valuable insights into different aspects of the segmentation accuracy and can collectively contribute to a thorough evaluation of a model’s effectiveness. The metrics, DSC, sensitivity, and specificity, are calculated using the following formulas:

(2) $$DSC=\frac{2TP}{2TP+FP+FN}$$

(3) $$Sensitivity=\frac{TP}{TP+FN}$$

(4) $$Specificity=\frac{TN}{TN+FP}$$

In these equations, TP represents true positives, which correspond to voxels correctly classified by the model. FP stands for false positives, FN represents false negatives, and TN denotes the number of true negatives.

## 4. Methodology

**Figure 3:**
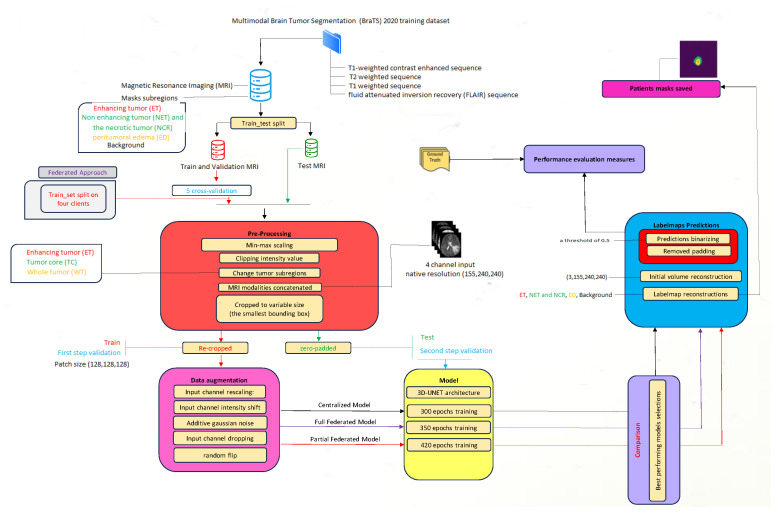
Overall block diagram of the proposed strategy used for enhancing brain tumor segmentation accuracy and preserving data privacy.

Our approach employs two distinct training pipelines with the same neural network architecture based on the 3D U-Net. We initially adopted a centralized approach to train the 3D model, utilizing a single-site dataset for model training and evaluation. Subsequently, we evaluated the FL using the same 3D U-Net architecture as the shared model, with a modified federated averaging (FedAvg) serving as the aggregation algorithm. The experiments used the BraTS2020 datasets, focusing on multiclass brain tumor segmentation. Within the FL pipeline, distributed devices perform segmentation tasks using their respective local data, ensuring no data sharing occurs among participants to uphold data privacy. The subsequent sections provide detailed insights into each pipeline and underscore the distinct effects of the privacy-preserving FL techniques employed. Comprehensive training configurations and other technical aspects of the proposed method are expounded. To provide a visual representation of our proposed methodology, Figure 3 illustrates a flowchart outlining the fundamental steps and components of our approach.

### 4.1. Federated Learning (FL)

FL revolutionizes traditional ML paradigms by prioritizing data privacy among collaborating institutions. Initially introduced in 2017 by Google [45], FL is characterized by a decentralized approach where a central server coordinates multiple clients in analyzing data using a shared model. Unlike conventional methods, FL eliminates the need for data transfer between devices, preserving the privacy of each client’s data. Instead, only model parameter updates are exchanged during training rounds.

#### 4.1.1. Federated Learning Types and Conditions

In general, FL can be categorized into three main types based on the distribution of client data: horizontal federated learning (HFL), vertical federated learning (VFL), and transfer federated learning (TFL). To elaborate, HFL represents an FL approach in which the datasets on the clients (i.e., devices) share the same feature space but contain different observations. VFL, also known as feature-based FL, is an approach where data from distinct domains are utilized to train the global model. In this scenario, the datasets for the clients may have the same observations but different features. Additionally, TFL, as another architecture, comes into play when the datasets on the devices differ in instances and features [27]. The potential of FL as a transformative technology is evident in its capacity to address many challenges. While FL techniques make noteworthy contributions to real-world problem solving, it is crucial to acknowledge and navigate a spectrum of constraints, encompassing [45,46]

- Non-IID (Non-Independently and Identically Distributed) Data: Patient data stored locally on different medical devices do not directly represent the entire population’s medical conditions.
- Unbalanced Local Data Sizes: Some medical institutions may have significantly larger datasets than others, introducing variations in the a available patient data.
- Massively Distributed: FL involves several medical institutions or clients participating in collaborative training.
- Limited Communication: When not all medical institutions are guaranteed to be online simultaneously, training may occur with a subset of devices, and the process may be asynchronous.

Medical institutions may have diverse patient populations with varying demographics, medical histories, and conditions. Therefore, treating the local data as representative of the entire population might lead to biased or inaccurate model training. Similarly, a large research hospital may have more extensive patient records than a smaller clinic. Addressing this unbalance is crucial to ensure fair model training and prevent biases toward institutions with larger datasets. Additionally, numerous hospitals, clinics, and research centers contribute their data for collective model training. This collaborative approach ensures a diverse representation of medical scenarios and conditions across the federated network. Furthermore, given the diverse and dynamic nature of healthcare settings, not all medical institutions may be online simultaneously for training. Therefore, training with flexibility in participation ensures that the FL process continues even when some institutions are temporarily offline. Overall, in medical FL, these challenges are particularly significant due to the sensitive nature of patient data. Ensuring that models are robust and generalizable across different medical institutions, regardless of variations in data distribution, size, and communication constraints, is essential for the successful and ethical application of FL in healthcare.

#### 4.1.2. Averaging Algorithms

A pivotal algorithm in FL is FedAvg, an extension of federated stochastic gradient descent [45]. In FedSGD, a client is randomly chosen to conduct a single batch gradient calculation during each communication round. The average gradient derived from its local data is then sent back to the server, where aggregation occurs, subsequently leading to the model update. On the other hand, FedAvg, introduces a variation wherein the client iteratively adjusts the weights, instead of the gradient, multiple times prior before transmitting them to the server for aggregation. FedAvg allows a network of clients to collectively train ML and DL models using their local data. This decentralized approach eliminates the need for clients to upload sensitive data to a central server, aligning with privacy requirements. The pseudocodes of FedAvg are provided in Algorithm 1.
**Algorithm 1** The FedAvg algorithm. K clients are indexed by k, B is the local minibatch size, E is the number of local epochs, and η is the learning rate.1:**Server executes:**2:initialize $w_{0}$3:**for** eachroundt=1,2,⋯ **do**4:     m←max(C·K,1)5:     $S_{t}\leftarrow$ (random set of *m* clients)6:     **for** $\mathrm{each}\mathrm{client}k\in S_{t}\mathrm{in}\mathrm{parallel}$ **do**7:          $w_{t+1}^{k}\leftarrow \mathrm{ClientUpdate}\left(k,w_{t}\right)$8:     **end for**9:     $w_{t+1}\leftarrow \sum_{k=1}^{K}\frac{n_{k}}{n}w_{t+1}^{k}$10:**end for**11:**ClientUpdate**(k,w):    //run on client *k*12:B← (split $P_{k}$ into batches of size *B*)13:**for** eachlocalepochifrom1toE **do**14:     **for** eachbatchb∈B **do**15:         w←w−η·∇l(w,b)16:     **end for**17:**end for**18:**return** *w* to Server

However, FedAvg exhibits challenges when using heterogeneous data, prompting research into alternative solutions. Notably, methods like FedAdam and FedAdagrad have been proposed to address these limitations and cater to specific scenarios. These alternatives aim to enhance FL’s performance, especially in the presence of diverse and heterogeneous datasets.

#### 4.1.3. Overall Architecture

**Figure 4:**
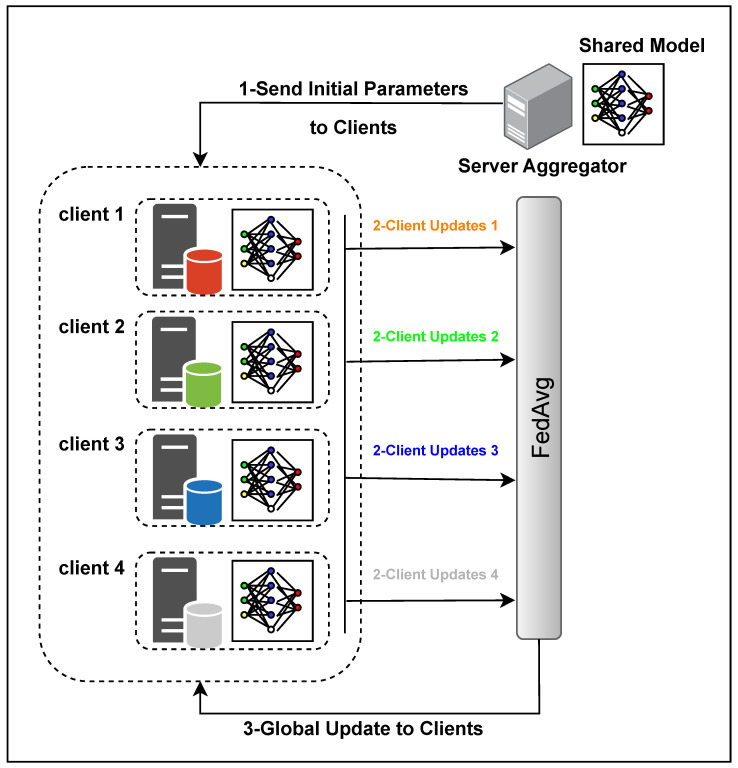
Federated learning topology.

FL operates on the principle of local training, model update aggregation, and parameter distribution, constituting a federated round. Hyperparameters like epochs per round (EpR), the number of participants, and model update compression/pruning methods [45] play crucial roles in FL’s success. The choice of EpR influences convergence, echoing the impact of learning rate and batch size in traditional training. Notably, challenges arise when datasets are non-IID and if the number of participants per round changes. In this study, we employed an FL approach with 3 epochs per round for 350 and 420 epochs. The training datasets consisted of 267 patient data points distributed unevenly among four clients with 37, 50, 80, and 100 data points, respectively. To simulate real-world scenarios, from epochs 50 to 150, we intentionally interrupted the connection with one random client out of the four participating clients every 10 epochs. As a result, the disconnected client was excluded from the averaging process with the remaining clients for that round. This deliberate disruption aimed to mimic network instability and client dropout, allowing us to assess the robustness of the FL framework under such conditions. Moreover, employing three epochs per round (EpR) mitigated diminishing returns. This is particularly crucial as we employed an HFL setup with non-IID datasets. The dynamic changes in the number of participants per round, coupled with the intricacies of performing 3D segmentation for all three tumor parts, contributed to the complexity of the task at hand. Figure 4 illustrates each round’s topology and steps involved in the FL model. Before training, clients establish connections with the server, which sends initial parameters to the clients. Clients then perform local training on their own data and send their updates to the server. The server employs the modified FedAvg technique to generate the new weights for the shared model, which is sent back to the participants for a new round. This iterative process continues until all rounds have been completed.

### 4.2. Privacy Preservation

In the pursuit of safeguarding sensitive information and ensuring user privacy, a comprehensive set of PP measures should be considered. These measures are designed to address various aspects of data security, user confidentiality, and system resilience. The following outlines the key components of the PP approach [34,38]:

- Encryption and Security
  - Objective: Implement robust encryption mechanisms to secure data during transmission and storage, preventing unauthorized access.
  - Methodology: Utilize advanced cryptographic techniques, including homomorphic encryption, and secure multiparty computation (SMPC) and blockchain to maintain the confidentiality of data.
- Differential Privacy
  - Objective: Ensure that the FL model does not reveal information about specific data points to protect individual data contributors.
  - Methodology: Introduce controlled noise or randomness to the learning process, preserving individual privacy while maintaining the utility of the model.
- Leakage Measures
  - Objective: Quantify and assess potential information leakage during the FL process.
  - Methodology: Evaluate the extent to which individual data points or model information might be unintentionally disclosed, employing leakage measures for comprehensive analysis.
- Communication Overhead
  - Objective: Minimize the amount of communication between the central server and participating clients to enhance privacy.
  - Methodology: Optimize communication protocols and reduce unnecessary data exchange, balancing the need for information transfer with privacy considerations.
- Security Analysis
  - Objective: Conduct a thorough security analysis to identify vulnerabilities and threats to the FL system.
  - Methodology: Assess the system’s robustness against potential attacks, ensuring the implementation of effective countermeasures.
- Threat Modeling
  - Objective: Anticipate potential threats to the privacy of the FL system.
  - Methodology: Develop models to understand and mitigate identified threats, aligning the privacy measures with the anticipated risks.
- User Perception
  - Objective: Consider end-user perceptions and expectations regarding privacy protection measures.
  - Methodology: Align PP mechanisms with user expectations, ensuring transparency and user acceptance.
- Trade-offs
  - Objective: Acknowledge and discuss trade-offs between privacy preservation and model performance.
  - Methodology: Evaluate the impact of privacy measures on model utility and find a balance that aligns with the overarching goals of the FL system.
- Evolving Attack Techniques
  - Objective: Stay informed about emerging privacy attack techniques.
  - Methodology: Continuously update FL system defenses to adapt to evolving threats, ensuring the system’s resilience against new attack vectors.
- Reward-Driven Approaches
  - Objective: Encourage participants to contribute data while protecting their privacy.
  - Methodology: Implement incentive structures that reward data contributors, striking a balance between participation encouragement and privacy preservation.

**Figure 5:**
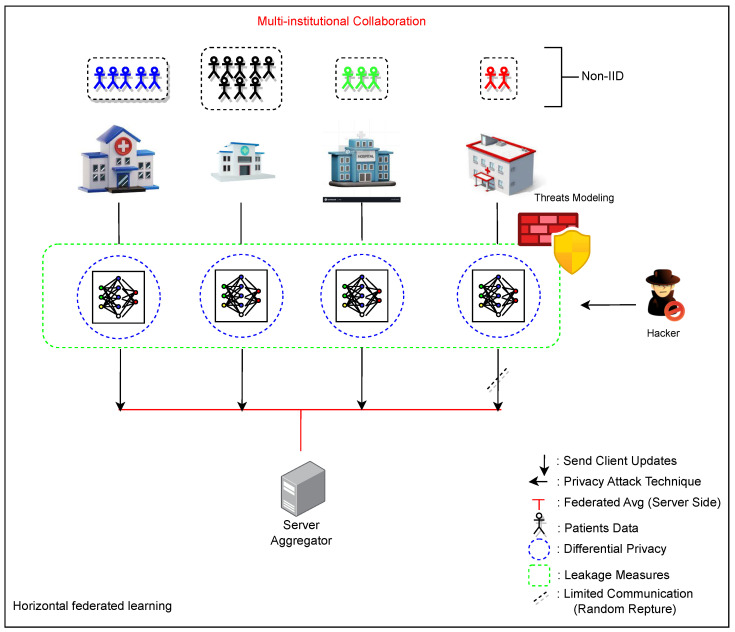
A comprehensive set of privacy-preserving measures.

In our FL approach, differential privacy is implemented through perturbation methods, incorporating randomly generated noises into each patient’s data point and for each client before the training process begins. Noise is introduced to local models as well, to improve privacy and to avoid accidental or intentional leakage of sensitive information. Additionally, data augmentation techniques are employed to introduce diversity. During training epochs, a shuffle technique is applied, which can also help to avoid overfitting and improve privacy by preventing the model from memorizing the order of the data points and the training data by introducing randomness into the training process. In this approach, a comprehensive security analysis is conducted to identify and address vulnerabilities, ensuring the robustness and integrity of our FL system against potential threats, guiding the development of effective countermeasures; see Figure 5.

Encryption and security measures, while crucial, are reserved for future works. To avoid the leakage of sensitive information during model sharing and to prevent potential adversaries from launching attacks, we carefully select a random subset of local models’ training weights to share during each federation round. This measure, combined with differential privacy, mitigates the risk of reverse attacks [47]. To address the high latency and limited bandwidth issues, communication between clients and the server is limited to 1 time per round (3 epochs), starting from the ninth epoch. This strategic approach serves to both reduce the number of communication rounds and simultaneously limit the size of transmitted messages, optimizing communication and computational overhead. Understanding user perception is paramount. We aim to motivate users to willingly share their data by ensuring that the FL system is flexible. Users have the option to share only parts of their models, enhancing their confidence in the system’s ability to protect their privacy. Striking a balance between aggressive privacy measures and model utility is crucial. We continuously assess and manage trade-offs to optimize both privacy and performance. While reward-driven approaches could be effective in real-life scenarios with clinics and hospitals, we currently focus on the PPTs mentioned above Section 4.2.

### 4.3. Privacy-Preserving Algorithm

**Figure 6:**
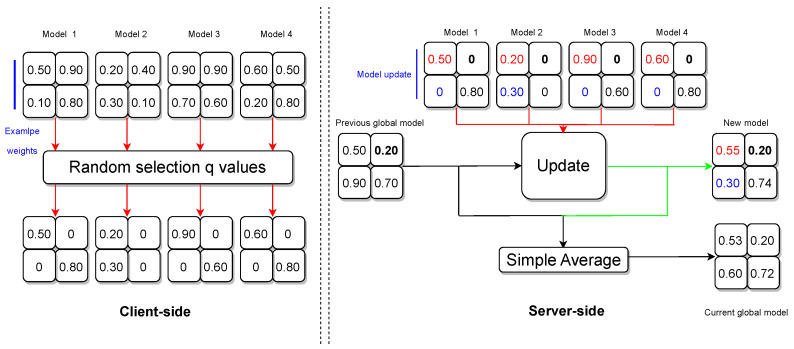
Federated learning privacy-preserving algorithm.

Inspired by [34], our PP algorithm was designed to enforce the protection of individual data contributors in FL. The FL model comprises 80 layers with diverse shapes, each containing numerous weights. The algorithm employs a selective approach, randomly choosing a fraction q of weights within each layer, while setting the nonselected weights to zero. For experimentation purposes, the q value, representing the proportion of selected weights, was randomly assigned between 0.4 and 0.5. A lower q value requires more computational resources and time to converge during the training process; see Figure 6.

During each federation round, clients transmit their local models to the aggregator to facilitate the update of global model parameters. On the server side, the algorithm scrutinizes each weight in every layer. The update process adheres to the following principles:

- Handling Zero Weights
  - If a weight is 0 for all clients, the algorithm retains the previous global model weight for that position in the new model.
  - If a weight is 0 for all clients except one, the algorithm incorporates the nonzero weight from the single client into the new model.
  - If weights are nonzero for a subset of clients, the algorithm computes the average of those nonzero weights and disregards clients with 0 weights.
  - If weights are nonzero for all clients, the algorithm computes the average of those weights.
- Global Model Update
  - The algorithm concludes by computing a simple average between the previous global model and the new model weights.
  - The adjusted server model is shared with all participating clients in preparation for the forthcoming federation round.

This PP algorithm ensures that sensitive information is carefully managed during the FL process, striking a balance between privacy preservation and model utility.

### 4.4. Training and Validation Details

We implemented a five-fold cross-validation strategy in the training phase to ensure robust model assessment. We separately trained the centralized model for each fold to identify the most effective one among the five folds. The final fold, the fifth one, was selected for its superior performance, taking into consideration time and resource constraints. Consequently, the FL model was exclusively trained on this chosen fold. Both pipelines utilized 3D pooling layers to downsample the input data. Each model was trained at least for 300 epochs, with a batch size of 1. Opting for a batch size of 1 enhanced training stability and ensured that the model adapted to individual instances, improving accuracy. The learning rate was fixed at 1 ×$10^{-4}$, and several optimizers were tested, such as Adam and Adamax. The Adam optimizer was chosen based on its superior performance, improving convergence and training results. Model performance was continuously assessed using the validation set during training. The optimal model was chosen based on the lowest loss value on the validation set; it was clear that this selected model would provide superior segmentation accuracy and generalization ability. The experiment was implemented in Keras and trained on an NVIDIA Tesla A100 40 GB GPU, provided via Google Colaboratory, manufactured by NVIDIA, Santa Clara, California, USA. Keras, known for its user-friendly interface, which facilitates seamless model development and extensive community support. The weight initialization technique used was ’he-normal’, particularly suitable for networks with rectified linear activation functions. This technique was used for both pipelines, the centralized model, and each model in the FL setup. We implemented a comprehensive two-step validation strategy to ensure model robustness and accuracy. In the first step, given the substantial size of the input image, we configured the input patch size to 128 × 128 × 128. This step was uniformly applied to each client model and the shared model, focusing exclusively on the validation set to monitor network performance during training continually. The second step involved additional validation for each pipeline. The initial volume underwent preprocessing steps similar to the training data, including cropping to the minimal brain extent. After the zero-padding operation, segmentation predictions were obtained for both the centralized and FL models, and any padding was removed. A threshold of 0.5 was applied to obtain binary predictions, and label map reconstruction involved boolean operations to delineate tumor regions accurately.

## 5. Results and Discussion

In this section, we present the results from the experiments on the Brats2020 dataset using the FL technique. To evaluate the performance of the FL approaches on multiclass segmentation, experiments were completed in both centralized and decentralized modes, encompassing both partial and full federated deep models. Each model is presented and discussed as a standalone scenario. Following this, we navigate the nuances of the partial federated model, where a random fraction of local model weights between 40 to 50% is shared among clients during the global federated process. This partial sharing approach is contrasted with the full federated model, wherein all local model weights are collaboratively fused. Each model was evaluated over a specific duration, with results presented regularly, offering a dynamic view of the training progression. To facilitate a meaningful comparison, a traditional centralized approach employing the same 3D U-Net architecture was consistently employed throughout, allowing us to gain insights into the relative efficacy of FL techniques. DSC, HD95, sensitivity and validation loss are used to visualize the difference in performance and productivity between the FL models and and their centralized counterparts. A modified FedAvg algorithm facilitates parameter aggregation, ensuring robust convergence during the FL process.

### 5.1. First Step Validation

#### 5.1.1. Partial Federated Deep Model

**Figure 7:**
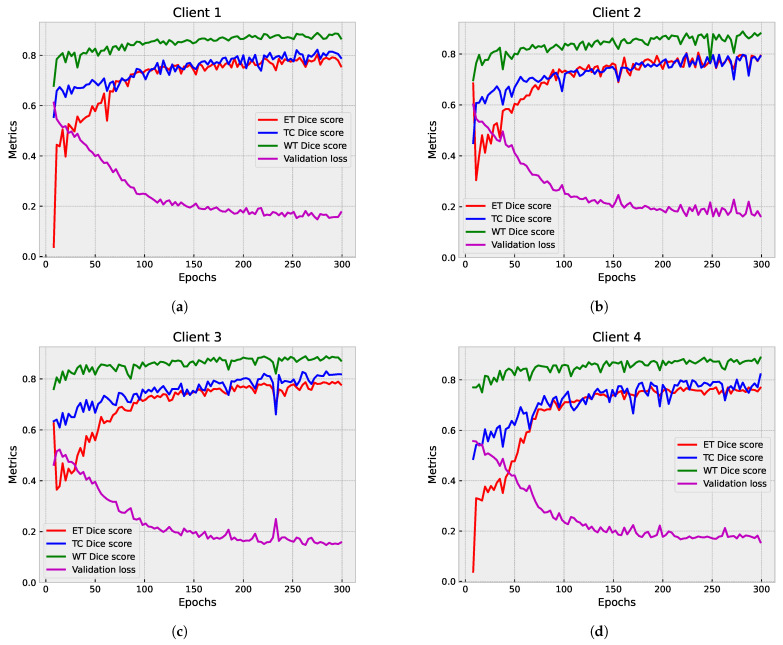
Partial federated mode analysis: examination of the segmentation results on the validation set, showcasing the contributions from each site. (**a**–**d**) display the ET, TC, and WT Dice scores, along with the validation loss for Clients 1 to 4, respectively.

**Figure 8:**
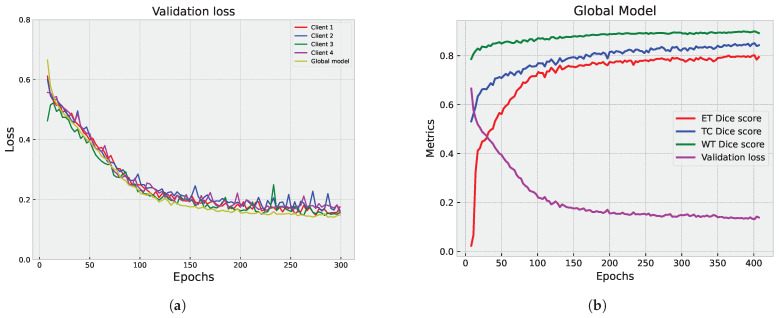
Partial federated mode analysis: validation loss and Dice score metrics. (**a**) Validation loss comparison: server vs. clients. (**b**) Dice score metrics’ plot: server aggregator performance.

The primary objective of the partial federated deep model is to fortify the protection of sensitive patients data. In Figure 7, we present a detailed examination of the segmentation results on the validation set, showcasing the individual contributions from each participating client. Concurrently, Figure 8a,b provide a comprehensive view of the aggregated results from the server. It is crucial to note that the results from individual clients serve an informational purpose, offering insights into their standalone performance. Given the limited number of clients (four in this instance), observing their individual performance becomes instrumental in identifying potential variations in the results.

Upon analysis of the results, a discernible trend emerged. The global model is stable and consistently performs well across epochs, with lower validation loss compared to individual clients. In contrast, the federated clients’ models, while achieving scores that are slightly lower than the global model, demonstrate a lack of stability, manifesting as numerous drops in all evaluated metrics, including ET Dice score, TC Dice score, WT Dice score, and validation loss. This observation underscores the intricate dynamics of the FL process and prompts a closer examination of the factors influencing the fluctuating performance of individual clients within the federated framework.

#### 5.1.2. Full Federated Deep Model

**Figure 9:**
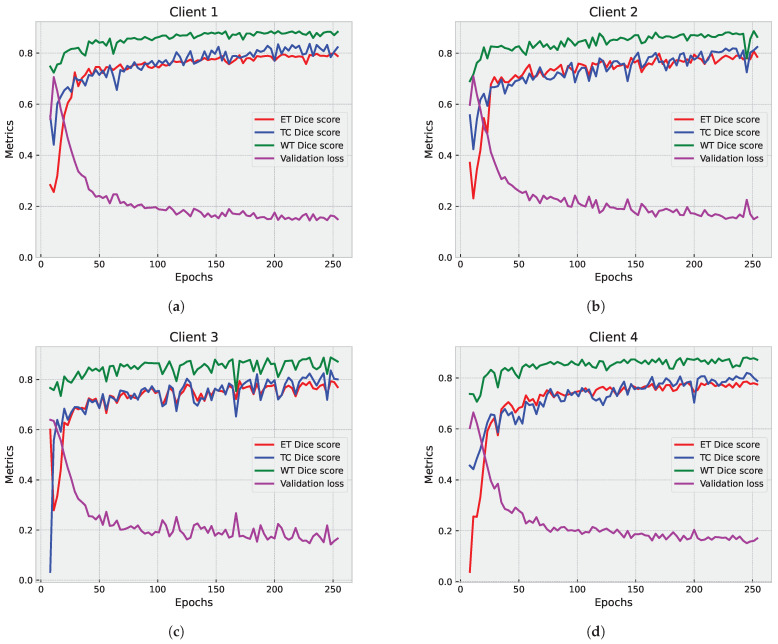
Full federated mode analysis: examination of the segmentation results on the validation set, showcasing the contributions from each site. (**a**–**d**) display the ET, TC, and WT Dice scores, along with the validation loss for Clients 1 to 4, respectively.

**Figure 10:**
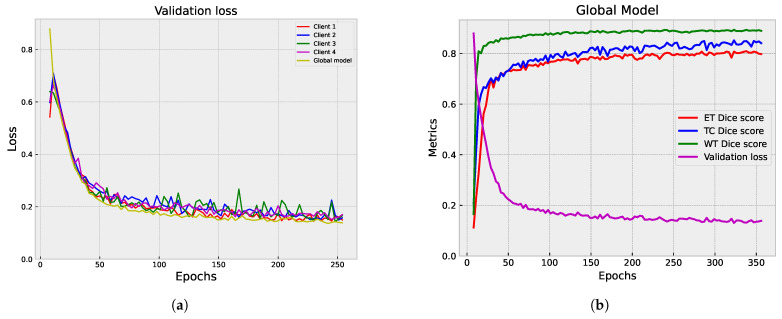
Full federated mode analysis: validation loss and Dice score metrics. (**a**) Validation loss comparison: server vs. clients. (**b**) Dice score metrics’ plot: server aggregator performance.

Figure 9 and Figure 10 showcase the outcomes of the segmentation process on the validation set. In Figure 9, the individual results from each participating client are displayed, providing valuable insights into how each client’s model performs on its own data.

Figure 10a,b illustrate the aggregated results from the server, offering a comprehensive view of the overall model performance. The global model demonstrates stability and consistent performance across epochs, achieving a lower validation loss compared to individual clients. While the federated clients’ models achieve competitive scores, they have slightly lower performance metrics than the global model. The individual client models show fluctuations and drops in different metrics, indicating variability in their performance.

#### 5.1.3. Centralized vs. Federated Approach: Convergence and Performance Analysis

**Figure 11:**
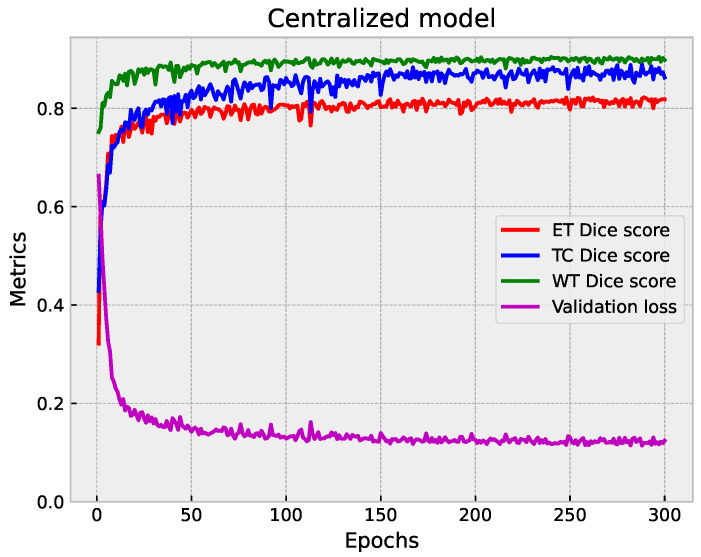
Plot of the validation loss with Dice score metrics of the centralized model.

We compared the performance of the centralized and federated models in brain tumor segmentation. The analysis focused on each approach’s convergence speed and effectiveness in achieving accurate segmentation results. Figure 11, Figure 8b and Figure 10b provide a visual representation of the training progression for each approach. Notably, the centralized model exhibits faster convergence, completing training in 300 epochs with validation performed at every epoch, reaching convergence around epoch 150. Conversely, both the full federated and partial federated models require 350 and 420 epochs, respectively, with validation conducted once every round (three epochs) starting from epoch nine, with convergence around epoch 250 and 350, respectively. In the FL setup, the training times vary across individual clients. Client 1’s model takes approximately 72 s for training per epoch, Client 2’s model requires around 100 s per epoch, Client 3’s training process takes about 154 seconds per epoch, and Client 4’s model exhibits longer training times, around 190 s per epoch.

**Figure 12:**
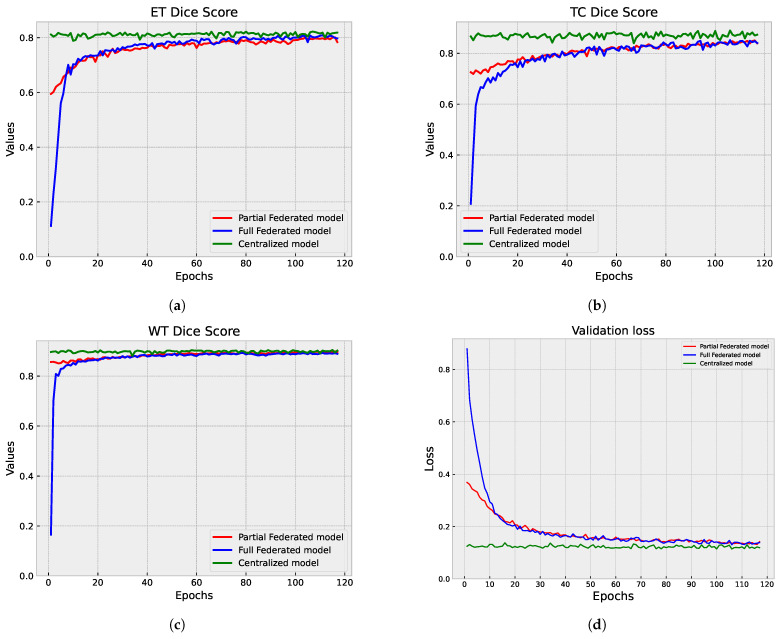
First step of validation comparison: centralized vs. partial federated vs. full federated approach. (**a**–**c**) Three cases of Dice score comparison, representing ET, TC, and WT, respectively. (**d**) Validation loss comparison.

On the server side, each federated round takes approximately 10 s for training and the same duration for validation (110 s). Therefore, completing each federated round requires approximately 2108 s (about 35 min). These detailed timings provide insights into the computational costs and resource requirements associated with the FL approach. A closer inspection of Figure 12, which illustrates the metrics of those models over their last 118 epochs, facilitates a comprehensive analysis and visualization. We observe that all models achieve a segmentation DSC above 80%. However, the centralized model demonstrates superior performance, as evidenced by a lower validation loss. Particularly noteworthy is the TC segmentation, where the centralized model attains values closer to 0.89. The federated models yield comparable segmentation results in terms of WT DSC, aligning closely with the centralized model. In the ET segmentation, the partial federated model trails slightly behind the full federated model, yet both experience a marginal drop of approximately 1% in performance compared to the centralized model. This nuanced analysis sheds light on the trade-offs between the efficiency of centralized training and the collaborative nature of FL, offering valuable insights into the model convergence dynamics and segmentation outcomes.

### 5.2. Second Step of Validation: Whole-Image Validation

**Figure 13:**
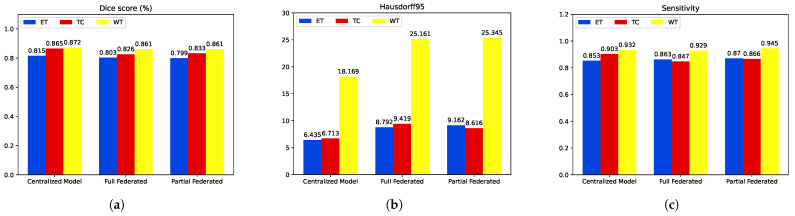
Second step of performance comparison: federated vs centralized learning. (**a**) Dices values. (**b**) Hausdorff distance. (**c**) Sensitivity.

Table 1 and Figure 13 show the results from the validation process. The centralized model performs strongly on whole-image validation, achieving high DSCs across all tumor types. The model exhibits low HD95s, indicating good boundary proximity. The sensitivity scores further confirm the model’s effectiveness in capturing true positives, with notable performance in TC and WT.

The partial federated model maintains commendable performance on whole-image validation, with competitive DSC and sensitivity values. Although slightly higher HD95s are observed than the centralized model, the model still captures relevant structures effectively. The full federated model maintains competitive performance, with good DSC and sensitivity values. Similar to the partial federated model, slightly higher HD95s are observed, but the model effectively captures tumor structures. Both federated models, despite a slight decrease in performance compared to the centralized model, demonstrate robust segmentation and sensitivity on whole-image validation, showcasing the potential of FL in preserving data privacy while achieving meaningful medical image segmentation results. The choice between partial and full federated models may depend on the specific privacy and collaboration requirements of the application.

### 5.3. Inference: Testing on Unseen Data

**Figure 14:**
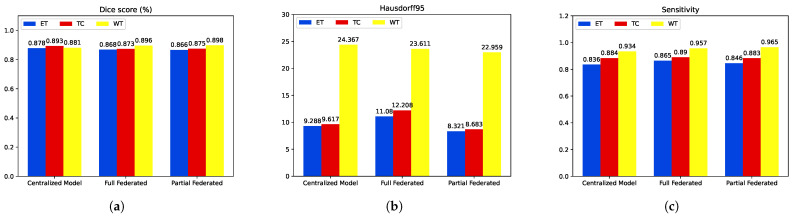
Inference performance comparison: federated vs centralized learning. (**a**) Dices values. (**b**) Hausdorff distance. (**c**) Sensitivity.

**Figure 15:**
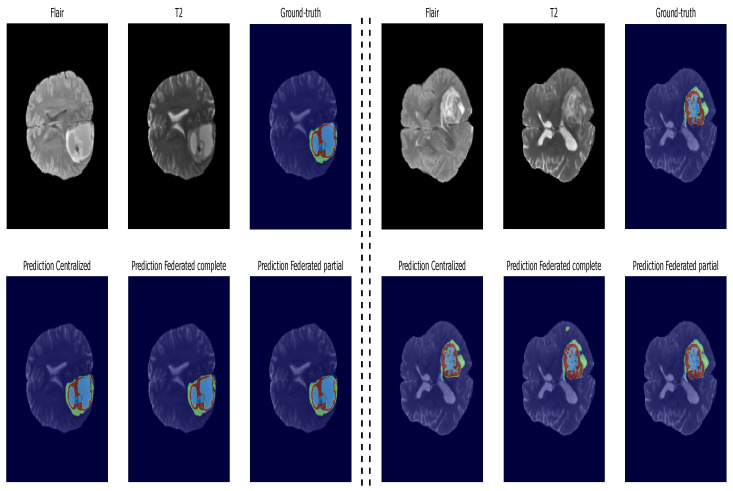
Visual segmentation results of proposed methods from two different patients’ data. Axial slice of MRI images in two modalities, ground truth, and predicted results from both the centralized and federated models.

The networks’ performance evaluation was carried out on unseen data. Table 2 summarizes the performance metrics of the proposed models, while Figure 14 presents bar plots, providing a better visualization. Upon closer examination, both federated models exhibit comparable segmentation results in terms of ET Dice score, with only a marginal 1% drop compared to the centralized model. Notably, the federated models demonstrate superior performance in WT segmentation, showcasing a 1.5% improvement over the centralized model. TC segmentation has a slight performance gap compared to the centralized model, with a 2% drop; it is worth noting that the federated models outperform the centralized model in terms of HD95, particularly the partial federated model, which exhibits the lowest values for all tumor parts compared to both the centralized and full federated models. Furthermore, regarding sensitivity, both federated models surpass the centralized model, indicating that the HFL system, with the modified FedAvg as the aggregating algorithm, excels in multiclass segmentation tasks. This nuanced analysis underscores the effectiveness of the FL approach, balancing privacy concerns with robust segmentation performance on diverse datasets. For example, Figure 15 displays segmented tumors from the test set. These visual representations offer a succinct overview of the models’ strengths and weaknesses, aiding in interpreting the nuanced results discussed above.

### 5.4. Model Comparisons with the State-of-the-Art Methods

In comparison to state-of-the-art centralized approaches that employ similar methodologies, our federated models demonstrate competitive performance on the test set. In Table 3, the performance evaluation of our federated models reveals their competitive edge over several existing methods. Drawing comparisons with the method of Liu et al. (2023) [18], our federated models showcase a superior DSC in the ET region, with a notable improvement of 6%. Similarly, in the TC regions, our models outperform those of Sahoo et al. (2023) [11] and Hu et al. (2023) [12] by 1.5% and 31%, respectively, demonstrating its efficacy in capturing core tumor structures. Furthermore, our federated models exhibit exceptional results for the WT subregion, surpassing the scores of Silva et al. (2021) [10] and Liu et al. (2023) [13] by 1% and 6.7%, respectively.

**Figure 16:**
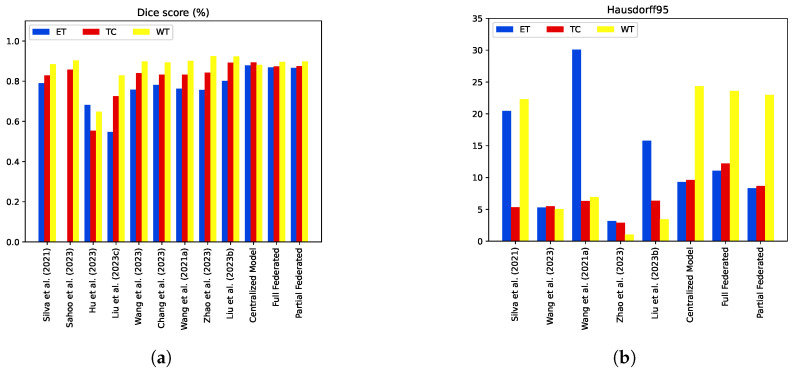
Performance comparison of proposed method with state-of-the-art techniques [10,11,12,13,14,15,16,17,18] on Brats2020 test set in terms of dice coefficient and Hausdorff distance. (**a**) Validation Dice values. (**b**) Validation Hausdorff distance.

Moreover, our federated models excel in boundary delineation, evident from the lower HD95 values across all tumor regions compared to several related methods. For instance, in the ET region, our partial federated model achieves an HD95 value of 8.3, outperforming the corresponding value of Liu et al. (2023b) (15.8) [18]. Additionally, our models demonstrate enhanced sensitivity values for all tumor regions, indicating superior tumor area detection compared to prior methods. These comparative results emphasize the efficacy of our federated models in achieving improved segmentation performance on the Brats2020 test set. The detailed metrics provided in Table 3 are visually represented in Figure 16 to provide comprehensive insights into the segmentation performance of our federated models.

Our results present a significant advance in the application of FL for brain tumor segmentation, particularly in the context of PP medical imaging. Our results not only contribute to the existing body of knowledge but also highlight the potential of FL to address critical challenges in healthcare. Our model achieved DSCs of 0.898, 0.875, and 0.866 for the WT, TC, and enhanced tumor core, respectively. These results are consistent with the performance measures reported in the recent literature, where DL models applied to the Brats dataset have demonstrated similar or slightly lower levels of performance. For example, studies using 2D and 3D convolutional neural networks reported DSCs ranging from 0.85 to 0.90, indicating that our approach is competitive and effective for accurately segmenting brain tumor regions. This performance is particularly noteworthy given the complexities inherent in brain tumor imaging, including variations in tumor morphology and the presence of surrounding anatomical structures. The integration of FL into our methodology represents a paradigm shift in how medical imaging data can be used for model learning. Traditional centralized approaches often face significant obstacles related to data confidentiality, security, and regulatory compliance. By employing FL, we enable model training on decentralized data sources, without the need to transfer sensitive patient information. This approach not only mitigates confidentiality issues but also enhances the potential for collaboration between institutions, enabling the sharing of diverse datasets while adhering to ethical standards. Our results build on previous research highlighting the benefits of FL in healthcare applications. For example, studies have shown that FL can improve model performance while preserving patient data privacy, which promotes confidence in AI applications in clinical settings. The successful implementation of differential privacy techniques in our study further highlights the importance of protecting individual patient data during the training process. This is essential as the risks of data leakage and model inversion attacks pose significant threats to patient privacy in traditional ML frameworks. Despite these promising results, our study has limitations. The performance of the FL model may be influenced by data heterogeneity between different institutions, including variations in imaging protocols and patient demographics. Future research should focus on expanding the dataset to encompass a wider range of tumor types and imaging modalities, enabling a more comprehensive assessment of the model’s performance. In addition, the exploration of advanced PPTs, such as homomorphic encryption and secure multiparty computation, could enhance the security of FL frameworks in medical applications. Furthermore, the development of the FL approach in real clinical environments has not yet been fully evaluated. Studying the practical implementation of FL in various healthcare environments will be essential to fully understand its feasibility and effectiveness in routine clinical practice.

## 6. Conclusions

In this research, we demonstrated the success of FL techniques in improving brain tumor segmentation from MRI images while prioritizing patient data privacy. Our analysis of partial and full federated deep models versus a centralized approach revealed that federated models achieved comparable segmentation performance, with notable advantages in terms of sensitivity and robustness, particularly in WT segmentation. The results obtained indicated that while the centralized model showed slightly superior performance metrics, the federated models maintained a high level of accuracy, with only marginal decreases in DSC for improved tumor segmentation and TC. Furthermore, the partial federated model outperformed its counterparts in terms of Hausdorff95 metrics, suggesting the better delineation of tumor boundaries, which is essential for clinical applications. This study highlights the potential of FL to facilitate collaborative research between institutions without compromising sensitive patient data, thereby removing an important hurdle in the field of medical imaging. The results support the wider adoption of FL methodologies in medical applications, paving the way for future research to explore more complex models and larger datasets, helping to improve patient outcomes and advance the field of medical imaging.

## Figures and Tables

**Table 1:** A comparative analysis of the performance of various approaches on the validation set. The metrics are presented as mean values. The highest score is indicated in bold.

Method	Dice			Sensitivity			Hausdorff95		
	ET	TC	WT	ET	WT	TC	ET	WT	TC
Proposed Centralized Model	0.815	**0.865**	0.872	0.853	0.932	**0.903**	6.435	18.169	6.713
Full Federated	0.803	0.826	0.861	0.863	0.929	0.847	8.792	25.161	9.419
Partial Federated	0.798	0.833	0.861	0.870	0.945	0.866	9.162	25.345	8.616

**Table 2:** Model performance results in our own test set extracted from the Brats2020 training dataset. Bold values represent the best performance for each metric.

Method	Dice			Sensitivity			Hausdorff95		
	ET	TC	WT	ET	WT	TC	ET	WT	TC
Proposed Centralized Model	**0.878**	**0.893**	0.881	0.836	0.934	0.884	9.28	24.36	9.61
Full Federated	0.868	0.873	0.896	**0.865**	0.957	**0.890**	11.088	23.611	12.208
Partial Federated	0.866	0.875	**0.898**	0.846	**0.965**	0.883	**8.321**	**22.959**	**8.683**

**Table 3:** Performance comparison of proposed method with state-of-the-art techniques on Brats2020 test set. The bold values indicate the best performance for each respective metric.

Method	Dice			Sensitivity			Specificity			Hausdorff95		
	ET	TC	WT	ET	WT	TC	ET	WT	TC	ET	WT	TC
[10]	0.79000	0.82970	0.88580	-	-	-	-	-	-	20.44	22.32	5.32
[11]	-	0.85750	0.90360	-	0.892	0.841	-	0.995	0.994	-	-	-
[12]	0.68250	0.55480	0.64820	-	-	-	-	-	-	-	-	-
[13]	0.54800	0.72620	0.82910	-	-	-	-	-	-	-	-	-
[14]	0.75800	0.84020	0.89910	-	-	-	-	-	-	**5.29**	5.07	5.51
[15]	0.78100	0.83200	0.89400	-	-	-	-	-	-	-	-	-
[16]	0.76380	0.83320	0.90100	-	-	-	-	-	-	30.09	6.96	6.30
[17]	0.75600	0.84300	**0.9240**	-	-	-	-	-	-	**3.19**	**1.04**	**2.88**
[18]	0.80200	**0.8920**	**0.9230**	-	-	-	-	-	-	15.80	3.44	6.35
Proposed Centralized Model	**0.878**	**0.893**	0.881	0.836	0.934	0.884	0.999	0.998	0.999	9.28	24.36	9.61
Full Federated	**0.868**	0.873	0.896	**0.865**	0.957	**0.890**	0.999	0.997	0.998	11.088	23.611	12.208
Partial Federated	**0.866**	0.875	0.898	0.846	**0.965**	0.883	0.999	0.998	0.999	**8.321**	22.959	8.683

## Data Availability

The data supporting the findings of this study are based on a publicly available dataset in the reference: Brats2020 at https://www.med.upenn.edu/cbica/brats2020/registration.html accessed on 20 November 2024.

## References

[1] Siegel R.L., Giaquinto A.N., Jemal A. (2024). Cancer statistics, 2024. CA A Cancer J. Clin..

[2] Litjens G., Kooi T., Bejnordi B.E., Setio A.A.A., Ciompi F., Ghafoorian M., van der Laak J.A., van Ginneken B., Sánchez C.I. (2017). A survey on deep learning in medical image analysis. Med. Image Anal..

[3] Bouamrane A., Derdour M. (2023). Enhancing Lung Cancer Detection and Classification Using Machine Learning and Deep Learning Techniques: A Comparative Study. Proceedings of the 2023 International Conference on Networking and Advanced Systems (ICNAS).

[4] Gasmi M., Derdour M., Gahmous A. (2022). Transfer learning for the classification of small-cell and non-small-cell lung cancer. Proceedings of the International Conference on Intelligent Systems and Pattern Recognition.

[5] Gasmi M., Derdour M., Gahmousse A., Amroune M., Bendjenna H., Sahraoui B. (2021). Multi-Input CNN for molecular classification in breast cancer. Proceedings of the 2021 International Conference on Recent Advances in Mathematics and Informatics (ICRAMI).

[6] Menaceur S., Derdour M., Bouramoul A. (2020). Using Query Expansion Techniques and Content-Based Filtering for Personalizing Analysis in Big Data. Int. J. Inf. Technol. Web Eng. (IJITWE).

[7] Mounir A., Adel A., Makhlouf D., Sébastien L., Philippe R. (2019). A New Two-Level Clustering Approach for Situations Management in Distributed Smart Environments. Int. J. Ambient. Comput. Intell. (IJACI).

[8] Kahil M.S., Bouramoul A., Derdour M. (2022). GreedyBigVis–A greedy approach for preparing large datasets to multidimensional visualization. Int. J. Comput. Appl..

[9] Kahil M.S., Bouramoul A., Derdour M. (2021). Multi Criteria-Based Community Detection and Visualization in Large-scale Networks Using Label Propagation Algorithm. Proceedings of the 2021 International Conference on Recent Advances in Mathematics and Informatics (ICRAMI).

[10] Silva C.A., Pinto A., Pereira S., Lopes A., Crimi A., Bakas S. (2021). Multi-stage Deep Layer Aggregation for Brain Tumor Segmentation. Brainlesion: Glioma, Multiple Sclerosis, Stroke and Traumatic Brain Injuries.

[11] Sahoo A.K., Parida P., Muralibabu K., Dash S. (2023). An improved DNN with FFCM method for multimodal brain tumor segmentation. Intell. Syst. Appl..

[12] Hu J., Gu X., Wang Z., Gu X. (2023). Mixture of calibrated networks for domain generalization in brain tumor segmentation. Knowl. -Based Syst..

[13] Liu Z., Wei J., Li R., Zhou J. (2023). Learning multi-modal brain tumor segmentation from privileged semi-paired MRI images with curriculum disentanglement learning. Comput. Biol. Med..

[14] Wang Y., Chen J., Bai X. (2023). Gradient-assisted deep model for brain tumor segmentation by multi-modality MRI volumes. Biomed. Signal Process. Control.

[15] Chang Y., Zheng Z., Sun Y., Zhao M., Lu Y., Zhang Y. (2023). DPAFNet: A Residual Dual-Path Attention-Fusion Convolutional Neural Network for Multimodal Brain Tumor Segmentation. Biomed. Signal Process. Control.

[16] Wang Y., Cao Y., Li J., Wu H., Wang S., Dong X., Yu H. (2021). A lightweight hierarchical convolution network for brain tumor segmentation. BMC Bioinform..

[17] Zhao J., Xing Z., Chen Z., Wan L., Han T., Fu H., Zhu L. (2023). Uncertainty-aware multi-dimensional mutual learning for brain and brain tumor segmentation. IEEE J. Biomed. Health Inform..

[18] Liu Z., Ma C., She W., Wang X. (2023). TransMVU: Multi-view 2D U-Nets with transformer for brain tumour segmentation. IET Image Process..

[19] Mohammed B.A., Senan E.M., Alshammari T.S., Alreshidi A., Alayba A.M., Alazmi M., Alsagri A.N. (2023). Hybrid techniques of analyzing mri images for early diagnosis of brain tumours based on hybrid features. Processes.

[20] Senan E.M., Jadhav M.E., Rassem T.H., Aljaloud A.S., Mohammed B.A., Al-Mekhlafi Z.G. (2022). Early diagnosis of brain tumour mri images using hybrid techniques between deep and machine learning. Comput. Math. Methods Med..

[21] Mohammed B.A., Senan E.M., Al-Mekhlafi Z.G., Rassem T.H., Makbol N.M., Alanazi A.A., Almurayziq T.S., Ghaleb F.A., Sallam A.A. (2022). Multi-method diagnosis of CT images for rapid detection of intracranial hemorrhages based on deep and hybrid learning. Electronics.

[22] Sheller M.J., Reina G.A., Edwards B., Martin J., Bakas S. (2019). Multi-institutional deep learning modeling without sharing patient data: A feasibility study on brain tumor segmentation. Proceedings of the Brainlesion: Glioma, Multiple Sclerosis, Stroke and Traumatic Brain Injuries: 4th International Workshop, BrainLes 2018, Held in Conjunction with MICCAI 2018.

[23] Qiu L., Cheng J., Gao H., Xiong W., Ren H. (2023). Federated semi-supervised learning for medical image segmentation via pseudo-label denoising. IEEE J. Biomed. Health Inform..

[24] Elbachir Y.M., Makhlouf D., Mohamed G., Bouhamed M.M., Abdellah K. (2024). Federated Learning for Multi-institutional on 3D Brain Tumor Segmentation. Proceedings of the 2024 6th International Conference on Pattern Analysis and Intelligent Systems (PAIS).

[25] Xu X., Deng H.H., Gateno J., Yan P. (2023). Federated multi-organ segmentation with inconsistent labels. IEEE Trans. Med. Imaging.

[26] Liu Q., Chen C., Qin J., Dou Q., Heng P.A. Feddg: Federated domain generalization on medical image segmentation via episodic learning in continuous frequency space. Proceedings of the IEEE/CVF Conference on Computer Vision and Pattern Recognition.

[27] Agrawal S., Sarkar S., Aouedi O., Yenduri G., Piamrat K., Alazab M., Bhattacharya S., Maddikunta P.K.R., Gadekallu T.R. (2022). Federated Learning for intrusion detection system: Concepts, challenges and future directions. Comput. Commun..

[28] Lazzarini R., Tianfield H., Charissis V. (2023). Federated learning for IoT intrusion detection. AI.

[29] Wang W., He F., Li Y., Tang S., Li X., Xia J., Lv Z. (2023). Data information processing of traffic digital twins in smart cities using edge intelligent federation learning. Inf. Process. Manag..

[30] Zhou F., Liu S., Fujita H., Hu X., Zhang Y., Wang B., Wang K. (2024). Fault diagnosis based on federated learning driven by dynamic expansion for model layers of imbalanced client. Expert Syst. Appl..

[31] Chen J., Xue J., Wang Y., Huang L., Baker T., Zhou Z. (2023). Privacy-Preserving and Traceable Federated Learning for data sharing in industrial IoT applications. Expert Syst. Appl..

[32] Qi T., Wu F., Wu C., He L., Huang Y., Xie X. (2023). Differentially private knowledge transfer for federated learning. Nat. Commun..

[33] Shao J., Wu F., Zhang J. (2024). Selective knowledge sharing for privacy-preserving federated distillation without a good teacher. Nat. Commun..

[34] Li W., Milletarì F., Xu D., Rieke N., Hancox J., Zhu W., Baust M., Cheng Y., Ourselin S., Cardoso M.J. (2019). Privacy-preserving federated brain tumour segmentation. Proceedings of the Machine Learning in Medical Imaging: 10th International Workshop, MLMI 2019, Held in Conjunction with MICCAI 2019.

[35] Ziller A., Passerat-Palmbach J., Ryffel T., Usynin D., Trask A., Junior I.D.L.C., Mancuso J., Makowski M., Rueckert D., Braren R. (2020). Privacy-preserving medical image analysis. arXiv.

[36] Lu Y., Huang X., Zhang K., Maharjan S., Zhang Y. (2020). Blockchain and federated learning for 5G beyond. IEEE Netw..

[37] Xu G., Zhou Z., Dong J., Zhang L., Song X. (2023). A blockchain-based federated learning scheme for data sharing in industrial internet of things. IEEE Internet Things J..

[38] Sameera K.M., Nicolazzo S., Arazzi M., Nocera A., KA R.R., Vinod P., Conti M. (2024). Privacy-Preserving in Blockchain-based Federated Learning Systems. arXiv.

[39] Kaissis G.A., Makowski M.R., Rückert D., Braren R.F. (2020). Secure, privacy-preserving and federated machine learning in medical imaging. Nat. Mach. Intell..

[40] Wang Y., Su Z., Zhang N., Benslimane A. (2020). Learning in the air: Secure federated learning for UAV-assisted crowdsensing. IEEE Trans. Netw. Sci. Eng..

[41] Zhang C., Li S., Xia J., Wang W., Yan F., Liu Y. {BatchCrypt}: Efficient homomorphic encryption for {Cross-Silo} federated learning. Proceedings of the 2020 USENIX Annual Technical Conference (USENIX ATC 20).

[42] Zhang L., Xu J., Vijayakumar P., Sharma P.K., Ghosh U. (2022). Homomorphic encryption-based privacy-preserving federated learning in iot-enabled healthcare system. IEEE Trans. Netw. Sci. Eng..

[43] Henry T., Carré A., Lerousseau M., Estienne T., Robert C., Paragios N., Deutsch E. (2021). Brain Tumor Segmentation with Self-ensembled, Deeply-Supervised 3D U-Net Neural Networks: A BraTS 2020 Challenge Solution. Lect. Notes Comput. Sci. (Incl. Subser. Lect. Notes Artif. Intell. Lect. Notes Bioinform.).

[44] Ronneberger O., Fischer P., Brox T. (2015). U-net: Convolutional networks for biomedical image segmentation. Proceedings of the Medical Image Computing and Computer-Assisted Intervention–MICCAI 2015: 18th International Conference.

[45] Brendan McMahan H., Moore E., Ramage D., Hampson S., Agüera y Arcas B. Communication-efficient learning of deep networks from decentralized data. Proceedings of the 20th International Conference on Artificial Intelligence and Statistics, AISTATS 2017.

[46] Konečný J., McMahan B., Ramage D. (2015). Federated Optimization:Distributed Optimization Beyond the Datacenter. arXiv.

[47] Hitaj B., Ateniese G., Perez-Cruz F. Deep models under the GAN: Information leakage from collaborative deep learning. Proceedings of the 2017 ACM SIGSAC Conference on Computer and Communications Security.

